# Mathematical Reflections on Acupoint Combinations in the Traditional Meridian Systems

**DOI:** 10.1155/2012/268237

**Published:** 2012-05-31

**Authors:** Sven Schroeder, Susanne Epplée, Jianwei Zhang, Gesa Meyer-Hamme, Thomas Friedemann, Weiguo Hu

**Affiliations:** ^1^HanseMerkur Center for Traditional Chinese Medicine, University Medical Center Hamburg-Eppendorf, Martinistra*β*e 52, House O55, 20246 Hamburg, Germany; ^2^Group TAMS, Department of Informatics, Faculty of Mathematics, Informatics and Natural Science, University of Hamburg, 22527 Hamburg, Germany; ^3^World Federation of Acupuncture-Moxibustion Societies, Beijing, China

## Abstract

The meridian system is a systematic order of empirical knowledge functioning as a rational ground for a balanced treatment by combining meridians. In TCM theory, a continuous circulation of Qi through 12 meridians is postulated, described as the Chinese clock (CC). On this basis, combinations of meridians and acupoints had been described in historical writings. The most common is the interior/exterior system beside the neighbouring system, the opposite clock system, and three systems, developed out of the theory of the six stages. All of these represent symmetrical combinations, which were defined by the steps in the CC. We calculated the possible combinations that fit into the systematics of the historical descriptions, leading to 19 systems. Merging the data of the 19 systems, possible steps in the CC clock for balancing a meridian are 1, 2, 3, and 6. Step 4 is not possible. Step 5 is a combinatory possibility but has no widespread tradition except for activating the yin extraordinary vessels. These possibilities can be plotted on the CC as a powerful tool for daily practice. Only two meridians might be excluded as potentially balancing meridians, so it seems almost impossible to define noneffective acupuncture points as controls in clinical trials.

## 1. Introduction

The theory of Chinese medicine has its basis in the old Chinese tradition dividing one into two (yin and yang) or dividing one into three (Tai Yang, Yang Ming, Shao Yang or Tai Yin, Jue Yin, Shao Yin or front, back, side) [1]. The development of the Chinese meridian system has the same basis, putting empirical knowledge into an organized system, dividing the body surface into areas in an absolutely symmetrical way. It is very likely that the system was organized for numerical and symmetrical reasons to find a paired cyclic pedant to the odd cycle of the five phases [1]. Old manuscripts only describe 11 meridians [2–4], while later sources describe 12 meridians [4]. In Chinese Medicine the human organism is understood as being linked in an endless circulation of Qi throughout the meridians at the latest since the publication of the Nan Jing [5]. Most acupuncture textbooks describe the Qi of the body as flowing in a systematical circular and continuous way through 12 meridians. Detailed diagrams of the circulatory courses of the 12 meridians were described in the Song dynasty. The book Pictures of Circulatory Courses of Meridians and the Internal Organs* (Cunzhen Huanzhong Tu)* by Yang Jie had an important influence on later descriptions of the Chinese clock [6]. Putting the systematics of the meridian system into diagrams became more popular in the Ming dynasty and was described in detail in the Lei Jing Tu Yi in 1624 [7]. Under the influence of daoism, chronobiological treatments in relation to the Chinese clock like *Ziwu liuzhu fa* were developed [8].

Balancing Yin and Yang is a basic concept in Chinese medical theory [9, 10]. Throughout the whole tradition of Chinese medicine combinations or connections of meridians (or their corresponding organs) were postulated, leading to acupoint combinations [11]. Meridians are classified as yin or yang meridians. Lung (LU), spleen (SP), heart (HT), kidney (KI), pericardium (PC), and liver (LR) are defined as yin meridians, and large intestines (LI), stomach (ST), small intestines (SI), bladder (BL), triple energizer (TE), and gall-bladder (GB) are defined as yang meridians [12–17]. The aim of acupuncture treatment is considered to maintain a balance of yin and yang organs and meridians. Disease is understood as a loss of balance between the yin and yang energies, but a relative dysbalance can occur between yin meridians or between yang meridians as well [10, 18].

**Figure 1:**
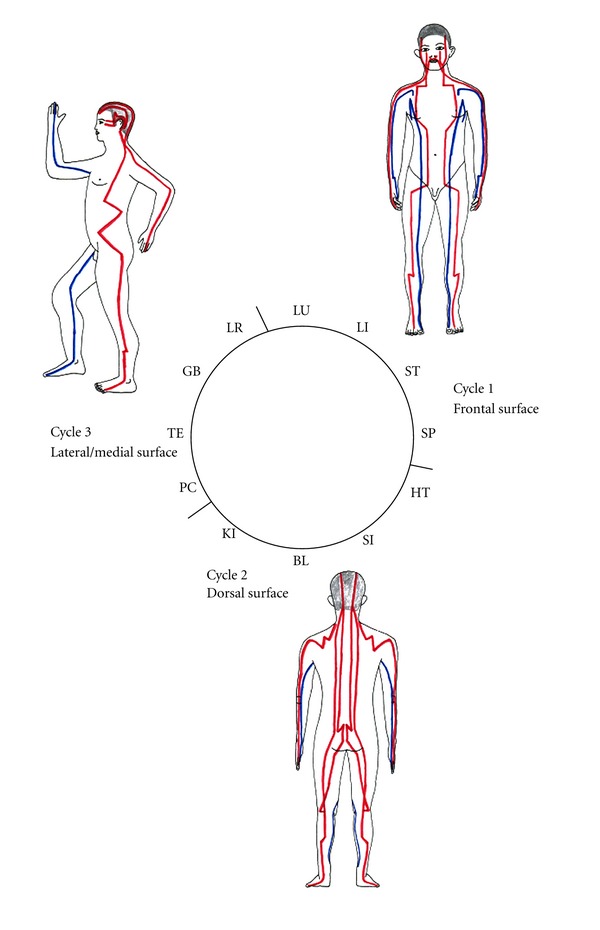
Connection of the three cycles in the Chinese clock with the body surface. Red: yang meridians; blue: yin meridians.

This circle of meridians in the Chinese clock is divided into three cycles, cycle 1 (LU, LI, ST, SP), cycle 2 (HT, SI, BL, KI), and cycle 3 (PC, TE, GB, LR) [19, 20]. A logical explanation for dividing the circle into three cycles is hard to find. We think there is a connection to the three human body surfaces by using an anatomical model of an upright standing person with the arms hanging down on the side, with the thumbs pointed frontwards and the 5th finger pointed toward the back. This is exactly the stance of most bronze models used for training acupuncture, especially since the redefinition and compilation of Wang Wei-yi, which was described in the Illustrated Manual on the Points for Acupuncture and Moxibustion on the New Bronze Model in about 1026 [21]. Cycle 1 can be connected to the frontal part of the body, cycle 2 can be connected to the dorsal part of the body, and cycle 3 can be connected to the lateral/medial side of the body. By dividing the Qi-flow into three cycles, circulation of Qi through three body surfaces, the frontal, dorsal, and lateral/medial parts of the human body, makes most sense to us (Figure 1).

## 2. Material

### 2.1. Literature Research for the Historical Systems

To understand the logic of combination of meridians we investigated modern textbooks and historical writings of Chinese medicine for the description of the known systems for combination of acupuncture points [1, 5, 7, 9, 12–17, 21–33].

### 2.2. Analysis of the Historical Systems

**Figure 2:**
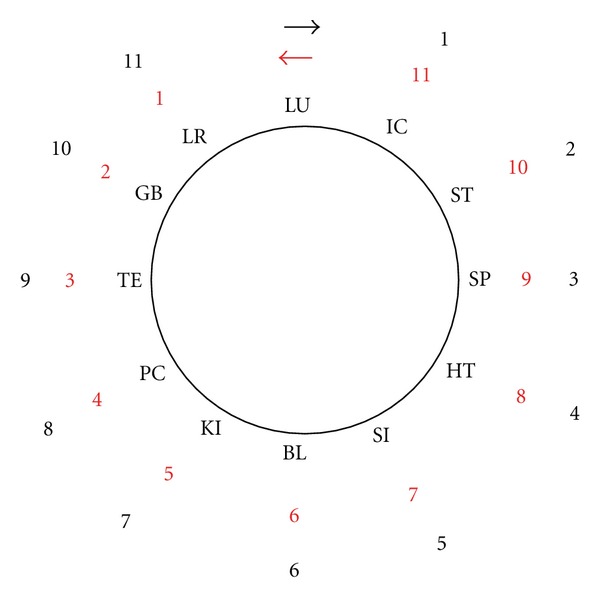
Counting of the steps in the Chinese clock, clockwise and counterclockwise.

1. First we described the steps taken in the Chinese clock in the different historical systems by counting clockwise and counterclockwise (Figure 2).

(2) Second we analysed the graphical pattern in the Chinese clock. Combining meridians in the Chinese clock leads to graphical patterns. We analysed whether these patterns have a certain rotational symmetry. An object has a rotational symmetry if it looks exactly the same at least once during a complete rotation through 360°. Rotation may be clockwise or counter clockwise. The angle at which an object looks exactly the same during rotation is called the angle of rotation. During the rotation, the object rotates around a fixed point, the centre of rotation, while its shape and size do not change. For example a full turn refers to a rotation of 360, a half turn refers to a rotation of 180, and a quarter turn refers to a rotation of 90°.
(3) Third we checked whether yin meridians were combined with yin meridians, yang with yang meridians, or yin with yang meridians.

### 2.3. Mathematical Analysis

To find out whether there are more systems than historically described and whether any meridians can be excluded as potentially balancing meridians, we calculated all symmetrical combinatorial possibilities.

Since there are 12 points in the Chinese clock, the smallest angle of rotation is 30°. It is not hard to imagine that 60° is the second possible angle of rotation since 60° is two times 30°. Of course, 90° is the third possible angle (a quarter of 360°). Next is 120° (one-third of 360°). But 150° is not possible since 360°cannot be exactly divided by 150°. The last one is 180°.

In summary, all possible rotation angles are 30°, 60°, 90°, 120°, and 180°.

## 3. Results

### 3.1. Description of the Historical Systems

#### 3.1.1. Interior/exterior (a Single-Step System)

The most common system is the interior-exterior connection. It originates from Chapter 2, Volume 1 of the Lingshu [29] and was described in detail in The Systematic Classic of Acupuncture and Moxibustion (Zhen jiu jia yi jing, Book 9) [23]. It is used in almost every ancient or modern school of Chinese medicine [23, 34–36]. It combines one meridian with the following one in the Chinese clock: LU with LI, ST with SP, HT with SI, BL with KI, PC with TE, and GB with LR. The meridians are next to each other on one extremity, connecting the exterior and the interior. There is always a yang (exterior)-yin (interior) combination (Figure 2(a)).

A typical example of combining interior and exterior points in a systematic way are the luo connecting points, as described in Chapter 10 of Lingshu, which can be combined with the Yuan-source points, following the interior/exterior combination system [9, 29].

Historical point combinations fitting into the interior/exterior system are LU-3 (Tianfu) and LI-4 (Hegu) for nose bleeding [28] or SP-1 (Yinbai) and ST-45 (Lidui) for nightmares [32], Jingman (GB 25) and Xingjian (LR-2) for lumbar pain, being not able to stand upright for a long time and bending back and forth [23].

#### 3.1.2. Neighbouring Channels (A Single-Step System)

This is the second option of combining channels in a single-step system, combining LR with LU, LI with ST, SP with HT, SI with BL, KI with PC, and TE with GB. It leads to arm-leg combinations of two yin or two yang channels [36–38].

Historical point combinations fitting in the neighbouring system are ST-36 (Zusanli) and LI-4 (Hegu) for dysenteric disorder [26] or PC-6 (Neiguan) and KI-6 (Zhaohai) for quick treating of abdominal disease [27]. 

#### 3.1.3. 6-Stage System I (a 1-Step–3-Step Alternating System)

This system of channel combination refers to the tradition dividing of a unit into 3 segments (Yang into Tai Yang, Yang Ming, and Shao Yang as well as Yin into Tai Yin, Jue Yin, and Shao Yin). It originates from the Suwen (Chapter 6, (77 + 79)) and Lingshu (Chapter 5, (948 + 949)) and describes 6 stages: Tai Yang, Yang Ming, Shao Yang, Tai Yin, Jue Yin, and Shao Yin [9, 29]. The sequence of the stages is in discussion. In the Suwen Chapter 6 the order described above can already be found as well as two other possibilities. Other authors of high influence like Zhang Zhongjing used an alternative order to the version described above in the Shanghanlun in the 2nd century [21], changing the order of Jue Yin and Shao Yin. The same order was used by Huang-Fu Mi in The Systematic Classic of Acupuncture and Moxibustion in the late 3rd century [23]. Since this time, meridians are named by the stages, Hand Tai-Yang (SI), Foot Tai-Yang (BL), Hand Yang Ming (LI), Foot Yang Ming (ST), Hand Shao Yang (TE), Foot Shao Yang (GB), Hand Tai Yin (LU), Foot Tai Yin (SP), Hand Jue Yin (PC), Foot Jue Yin (LR), Hand Shao Yin (HT) and Foot Shao Yin (KI). To our understanding the order described in Suwen (Chapter 6, (77+79)) and LingShu Chapter 5 (948 + 949)) is the most useful for daily practice, so we number the stages as described above. For understanding our theory of the development of the meridian system the numbering of the stages is of secondary importance and irrelevant for the central message of this paper.

Using the 6-stage theories for point combination is very common. The main system uses points connected to the same stage as the starting point. For this reason and because of the similar name (e.g. Foot and Hand Yang Ming) it is called “anatomical system” in some schools [39]. It combines Hand Tai Yang (SI) and Foot Tai Yang (BL), Hand Yang Ming (LI) and Foot Yang Ming (ST), Hand Shao Yang (TE) and Foot Shao Yang (GB), Hand Tai Yin (LU) and Foot Tai Yin (SP), Hand Jue Yin (PC) and Foot Jue Yin (LR), and Hand Shao Yin (HT) and Foot Shao Yin (KI). This system represents a 1-step–3-step alternating system. We call it the 6-stage system I.

Historical point combinations fitting into the 6-stage system I are PC-3 (Quze) and LR-13 (Zhangmen) for a dry mouth [32] or TE-5 (Waiguan) and GB-2 (Tinghui) for treating impaired hearing and deafness [29].

#### 3.1.4. **6-Stage System II (a 2-Step System)

The next system we call 6-stage system II. It can be seen as a development of 6-stage system I, because it combines Hand Tai Yin (LU) with Foot Yang Ming (ST) and Hand Yang Ming (LI) with Foot Tai Yin (SP), Hand Shao Yin (HT) with Foot Tai Yang (BL) and Hand Tai Yang (SI) with Foot Shao Yin (KI), Hand Jue Yin (PC) with Foot Shao Yang (GB), and Hand Shao Yang (TE) with Foot Jue Yin (LR). This system has a highly pragmatic relevance because it combines stages I and VI, stages II and IV, and stages III and V. It is widely used in modern schools [24, 37, 38] especially when diseases do not follow in the order of stages, but if there is stage hopping [35]. It uses two-step combinations, so we always get yin-yang or yang-yin combinations.

Historical point combinations fitting into this system are LU-7 (Lieque) and ST-36 (Zusanli) for acute dyspnoea [27] or LR-13 (Zhangmen) and TE-8 (Zhigou) plus TE-5 (Waiguan) for pain in the lateral costal region [30].

#### 3.1.5. 6-Stage System III (2-Step–6-Step Alternating System)

In the Ming-Dynasty Li Yan first described his “5-Zang extra relationship theory” [25]. Foot Tai Yang (BL) and Hand Tai Yin (LU), Hand Tai Yang (SI) and Foot Tai Yin (SP), Hand Jue Yin (PC) and Foot Yang Ming (ST), Foot Jue Yin (LR) and Hand Yang Ming (LI), Foot Shao Yang (GB) and Hand Shao Yin (HT), and Hand Shao Yang (TE) and Foot Shao Yin (KI) are combined. This system combines the yang meridians of stage I with the yin meridians of stage IV, the yin meridians of stage V with the yang meridians of stage II, and the yang meridians of stage III with the yin meridians of stage VI.

Historical examples using this system for point combinations are TE-6 (Zhigou) and KI-6 for constipation [26] or LI-4 (Hegu) and LR-3 (Taichong) for nasal polyps, nasal congestion, and discharge [27].

#### 3.1.6. Opposite Clock (6-Step System)

Cross needling in general was first described in Chapter 63 of Suwen. Applying this to the meridian circle is very popular and is called the opposite clock needling in one school [36] and is a basis for special techniques in Japanese acupuncture [24]. This system combines LU and BL, LI and KI, ST and PC, SP and TE, HT and GB, and SI and LR. Anatomically very distant areas of the human body are connected in a Yin-Yang combination.

Historical point combinations fitting into the 6-step system are LU-6 (Kongzui) and BL-31 (Shangliao) for febrile disease with an absence of sweating [23] or ST-8 (Touwei) and PC-7 (Dailing) for a splitting headache with severe eye pain [32].

#### 3.1.7. Common Features of the Historical Systems

**Figure 3:**
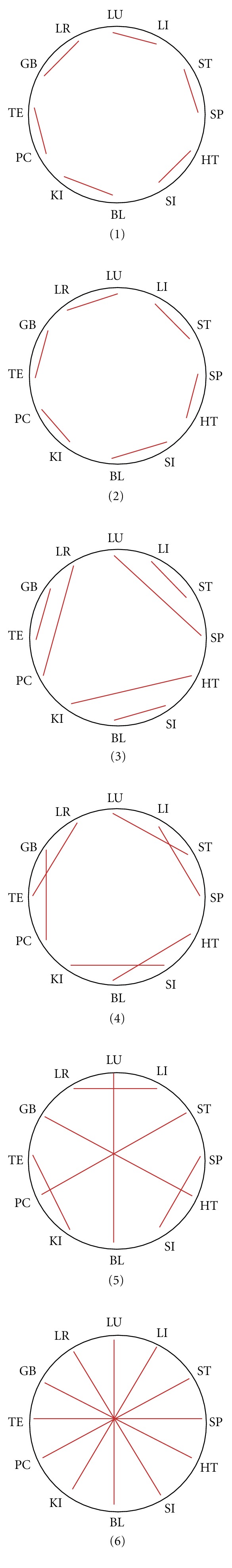
Graphical plotting of the historical systems: (1) interior/exterior; (2) Neighbouring channels; (3) 6-stage I; (4) 6-stage II; (5) 6-stage III; (6) opposite clock.

All historically described systems have in common that they build a symmetrical combination in the 12-meridian circle with a rotation symmetry of 30°, 60°, or 120°. Every meridian pair with only one other and no meridian is left over, so there are always 6 pairs of meridians. A maximum of two alternating steps are used, leading to yin-yang/yang-yin or to yin-yin/yang-yang combinations. They can be described as intrinsic rules of the historical systems, summarized in Table 1. A graphical plotting of all historical systems is shown in Figure 3. 

### 3.2. Graph Traversal Search for Further Systems

The 12 meridians in the circle were labelled 1, 2, 3,…, 12. They were divided into six groups. Every meridian pair with only one other and no meridian is left over. The possible combinatorial number is

(1) $$\frac{C_{12}^{2}\cdot C_{10}^{2}\cdot C_{8}^{2}\cdot C_{6}^{2}\cdot C_{4}^{2}}{\left(6!\right)}=\frac{7484400}{720}=10395,$$

where *C*_*n*_^*k*^ is the number of combinations of *n* things taken *k* at a time: *n*!/((*n*–*k*)!  *k*!)*k*! means the product of all the integers from 1 to *k*, that is, factorial.

Then, the steps of each group are calculated.

There are 6 possible steps in the Chinese clock: 1, 2, 3, 4, 5, and 6. (e.g., step 1 is a connection of two neighbouring meridians, step 3 skips one, combining the first meridian with the third one, etc.)

The number of possible combinations was counted. If the classification is rotationally symmetrical, it must satisfy the following requirements: the number of the steps is not 1, 3 or 5. The possible number is 1355. (We listed all possibilities by plotting the combinations using MATLAB.)

 At this stage we removed the third step combinations. In fact, we removed all the odd step numbers, that is, steps 3 and 3. But this situation satisfies the rotationally symmetrical rule, so in the next stage we will add it to the final results.

If the number of the step is 3 and 3, for example, all the steps are 1, 3, 1, 3, 1, 3, the number of step 1 is 3 and the number of step 3 is also 3. The possible combinatorial number satisfying the above situation is

(2) $$C_{6}^{2}\cdot C_{6}^{3}=15*20=300.$$

All combinations were plotted by MATLAB, and the symmetry was manually validated. 350 combinations showed a symmetrical pattern, of those one had 30°, 6 had 60°, 12 had 90°, 20 had 120°, and 311 had 180° rotation symmetry.

19 of the symmetrical patterns followed the intrinsic rules of the historical systems. One showed a rotation angle of 30°, 6 of 60°, none of 90°, 12 of 120°, and none of 180°. The steps in the Chinese clock were 1 step in 2 patterns, 1 step-3 steps alternating in 4 patterns, 2 steps in 4 patterns, 2 steps-6 steps alternating in 4 patterns, 3 steps in 2 patterns, 5 steps-7 steps alternating in 2 patterns, and 6 steps in one pattern (Table 2).

### 3.3. Additional Systems Found by Mathematical Calculation

#### 3.3.1. Step Systems

There was no additional system found beside the historical systems, interior/exterior and neighbouring channel systems.

#### 3.3.2. 1-Step-3-Step Alternating Systems

There are three more 1-step–3-step alternating systems. The first combines LU and LI, LR and ST, HT and SI, SP and BL, PC and TE, and KI and GB. The second combines LR and LU, GB and LI, SP and HT, ST and SI, KI and PC, and BL and TE. The third combines GB and LR, TE and LU, ST and SP, LI and HT, BL and KI, and SI and PC. They lead to additional point combinations not covered by those historically described.

#### 3.3.3. 2-Step Systems

There are three more 2-step systems. The first combines LU and GB, LI and LR, ST and HT, SP and SI, BL and PC, and KI and TE. The second combines LU and GB, LI and SP, ST and HT, SI and Kidney, BL and PC, and TE and LR. The third combines LU and ST, LI and LR, SP and SI, HT and BL, KI and TE, and PC and GB. They do not offer additional combinations of meridians.

#### 3.3.4. 2-Step–6-Step Alternating Systems

**Figure 4:**
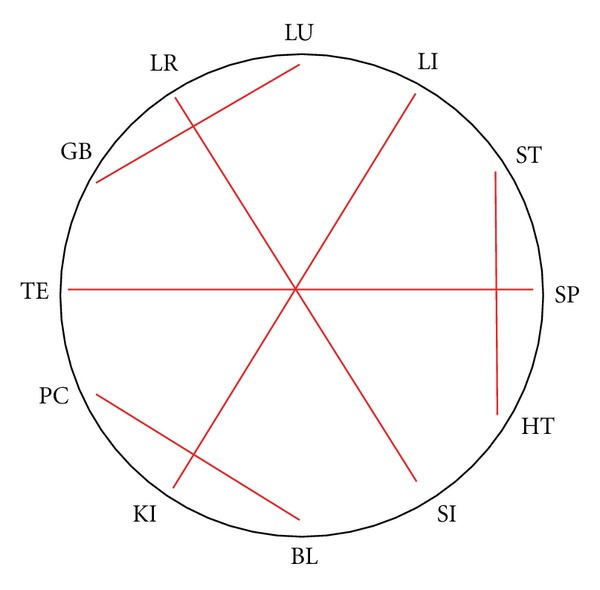
6 stage system IV.

There are three more 2-step–6-step alternating systems. The first is combining Hand Tai Yin (LU) and Foot Shao Yang (GB), Foot Tai Yang (BL) and Hand Jue Yin (PC), Foot Tai Yin (SP) and Hand Shao Yang (TE), Hand Yang Ming (LI) and Foot Shao Yin (KI), Hand Tai Yang (SI) and Foot Jue Yin (LR), and Foot Yang Ming (ST) and Hand Shao Yin (HT). It combines stages I and V, II and VI, and III and IV. We call it 6-stage system IV. Historical combinations fitting into the 6-stage system IV are ST-36 (Zusanli) and HT-8 (Shaofu) for difficult urination or retention of urine [32] or LU-7 (Lieque) and GB-12 (Wangu) for deviations concerning the mouth and face [31] (Figure 4).

The second 2-step–6-step alternating system combines LU and ST, LI and KI, SP and TE, HT and BL, SI and LR, and PC and GB. The third combines LU and BL, LI and SP, ST and PC, HT and GB, SI and KI as well as TE and LR. The second and the third 2-step–6-step alternating systems do not offer additional combinations.

#### 3.3.5. 3-Step Systems

There are two 3-step systems that follow the systematics of the intrinsic historical systems. They do not offer new combinations of meridians, because they are already covered in the 1-step–3 step alternating systems as shown above.

#### 3.3.6. 5-Step–7-Step Systems

Step 5 is a combinatorial possibility but has no large tradition in Chinese medicine except for concepts connected to the extraordinary vessels. The extraordinary vessels were not described in the Suwen or Lingshu as a system, but references can be found to the ren mai, chong mai, and qiao mai [9]. In the Nan jing the first written description of the extraordinary vessels in an organized summary can be found [39]. Treatment of the extraordinary vessels became more popular in the Jin-Yuan dynasty [22] and in the Ming dynasty [40]. The selection of four pairs of points, namely SP-4 and PC-6, LU-7 and KI-6, SI-3 and BL-62, and GB-41 and TE-5 refers to the *Zhen Jing Zhi Nan *written about 1295, which does not clearly mention the extraordinary vessels in the context of these four pairs of points [22].

In fact the first mention of therapy for the eight extraordinary vessels using the above described eight so called master points appeared in 1439 in the Complete Collection of Acupuncture and Moxibustion (Zhen Jiu Da Quan) by Xu Feng [40].

The eight master points are clearly described and connected to the extraordinary vessels and ordered in pairs: Chong mai (SP-4) and Yin Wei Mai (PC-6), Ren Mai (LU-7) and Yi Qiao Mai (KI-6), Du Mai (SI-3) and Yang Xiao mai (BL-62), and Dai Mai (GB-41) and Yang Wei Mai (TE-5) [41].

One often used technique to treat a disease connected to one of the extraordinary vessels is to combine the master points of the paired extraordinary vessels. Similar instructions are given in the Zhen Jiu Ju Ying [42], and, in Zhe Jiu Da Cheng [30], the master point of the treated extraordinary vessel is needled first, then the master point of the paired extraordinary vessel, called the coupled point, is needled second.

**Figure 5:**
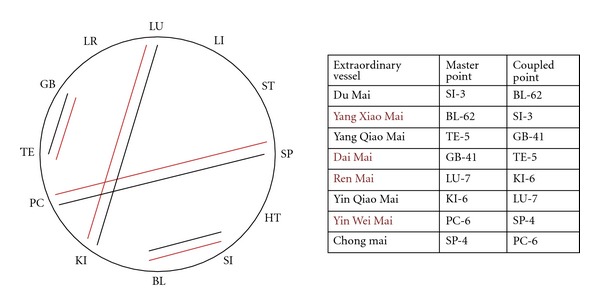
Activation of the extraordinary vessels (Qi jing ba mai) by combination of the master point and the coupled point.

All pairs of Yang extraordinary vessels are opened by a 1-step combination, already familiar from the 6-stage system I (see above), the Yin extraordinary vessels are opened by a 5-step (5-step–7-step, resp., if you consider a one directional flow in the Chinese clock) combination (Figure 5).

#### 3.3.7. 6-Step System

There is no additional system beside the opposite clock system.

### 3.4. Summary of the Results

The possibilities for finding a balanced treatment strategy can be described by the steps that have to be taken in the Chinese clock to combine acupuncture points. Merging the data of all systems, the steps in the Chinese clock showing a possibility for balancing are the following. Step 1, 2, and 3 are possible as well as Step 6. Step 4 is not a combinatorial possibility. Step 5 is a combinatorial possibility but has no tradition in TCM, except in the theory of the extraordinary vessel. A summary is given in Table 3. In addition, the affected meridian itself can be treated. In practice, when the affected (painful) area has been identified, (sensitive) ashi points on one or several of the chosen associated meridians can be found in correlated regions to the affected area. Pain can be reduced instantly by needling these ashi points [36].

**Figure 6:**
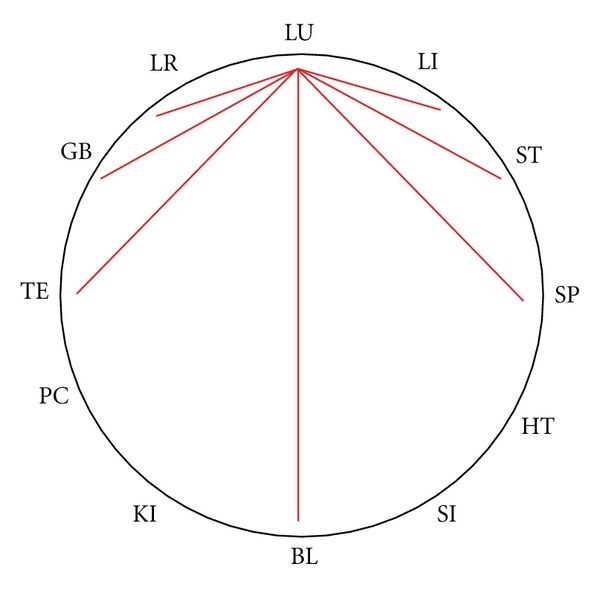
Merging of the combinatorial possibilities. (This can be done with every meridian in a similar way.)

Plotting the merged data of all 19 systems on the Chinese Clock, we found a tool for quick memorization. An example for the possible balancing meridians for the meridians of the lung is shown in Figure 6.

## 4. Discussion

Our work is based on an analysis of the historically described balancing systems. Seeing the theory of Chinese medicine as an inherently logical system, we used a mathematical approach to calculate the theoretical options based on the historical systems. These findings suggest that there are many more treatment options than normally expected.

The historically established systems for combining meridians cover many, but not all, of our calculated combinations of meridians. Our findings imply that every meridian can be balanced by possibly at least seven other meridians (and itself). Possible steps of balancing one meridian with another are the steps 1, 2, 3, and 6 in the Chinese clock. Step 4 does not offer a symmetric combinatorial option. Additionally step 5 is a mathematical possibility but has historically only a tradition in the theory of the extraordinary vessels (qi jing ba mai). For the opening of the yin extraordinary vessels a combination of step 5 and 7 is postulated [40]. The historical physicians might have thought that combining two odd steps in the Chinese clock and using a combination of anatomically very distant points is something exceptional. This might be one explanation why the historical physicians saw the necessity of the development of an extra theory [22, 39, 40].

Some schools of Chinese medicine devote more attention to describing systems developed from the systematics of the 6 stages [21], some schools combine mainly the opposite meridians of the 12-meridian circle [24], and some schools concentrate on combining all known systems [36–38]. These historically described systems must either be results of daily experience or have the advantage of fitting other theoretical approaches better. All these systems have been proved effective in daily practice by acupuncturists all over the world and are used with success.

Nevertheless, all these approaches are based on theoretical considerations from historical writings, supported by empirical knowledge obtained over the centuries as well as personal experience of the members of the schools. There are no controlled studies with hard data on the effect of point or meridian combinations so far.

The approach of this work, a mathematical search for further combinatorial possibilities, is as well a theoretical approach in the first line. It is based on the historical systems and a distinct order of the meridians in the Chinese clock.

As every meridian has the same power, a change of the order of the meridians will not change the number of mathematical possibilities for combination. While the order in the Chinese clock is not only accidental, but most likely selected for anatomical reasons (Figure 1), a different order in the Chinese clock presumably will not lead to successful treatment strategies.

Even though the historical systems have been used for centuries, from the theoretical point of view the newly discovered possibilities we found must have a similar power, because there are no superior meridians in the Chinese clock.

One of the newly discovered systems has to be emphasised. Like the above described three historical combinations, the 6-stage systems I, II, and III, it offers an extra opportunity to connect meridians of the 6 stages. It combines the meridians of stage I and V, II and VI, and III and IV in a combination of Yin-Yang and Yang-Yin, respectively. It might increase the view of how stages can follow and influence one another. We call it 6-stage system IV.

Broad research failed to uncover any mention in the literature on Chinese medicine so far. It might be a missing piece of the puzzle and could complete the view on the theory of the 6 stages.

While the authors of this paper have started to transfer their consideration into treatment strategies with remarkable success, their reflections still have only a theoretical basis and have to be approved by clinical studies.

Most controlled acupuncture trials used minimal, superficial, sham, or “placebo” acupuncture as control. Often points at locations distant from acupuncture points described in the textbook were used [43]. Only step 4 in the Chinese clock does not open any combinatorial possibility, so these meridians might function as theoretical areas for placebo treatment. But these meridians are anatomically extremely close to the affected or the unbalanced meridian. For example, if you look at the LU meridian, the meridian of the HT and PC is step 4 in the Chinese clock (the LU, PC, and HT meridians are direct neighbours on the arm). And if the ST meridian is chosen, GB and BL are step 4 in the Chinese clock (ST, GB, and BL are direct neighbours on the leg). This leads to the problem, considering that the peripheral nerves are involved in the effects of acupuncture, that anatomical variants of the peripheral nerves have an influence on the location of acupuncture points [44]. Tao showed that meridians are not clearly described in historical texts and that the localisation of acupuncture points in historical writings differs in some points extremely from descriptions in modern acupuncture textbooks [45].

In some patients painful affections can be found not only on a defined meridian area but also in regions between meridians. In these cases, pain-releasing ashi points are often needled between associated meridians, which was already described and discussed in the Systematic Classic of Acupuncture and Moxibustion [23] and is consistently used in some modern schools [36, 46, 47]. Additionally we can learn from acupuncture microsystems and related techniques, for example, Chinese and French ear acupuncture, Korean hand acupuncture, hand and foot reflexology, that other somatotopies exist which partly overlap with the classical meridian system. In consequence, defining anatomical regions without any influence on other regions seems extremely difficult. The use of needle insertions at locations distant from acupuncture points defined in acupuncture textbooks as controls in clinical trials is not suitable as an inert placebo [48].

Increasing the number of treatment options does not mean that all treatment strategies have similar power, but it shows that manipulation in multiple areas of the body surface can be potentially effective in acupuncture treatment. Generally a case can be solved successfully by different treatment strategies, but it is good to know which treatment is not very likely to solve a problem or could do some harm to the patient. Knowing different treatment possibilities is especially helpful in pain management, where usually a fast effect is needed for quick pain relief. There might be an area of repletion (fullness) on the body surface which helps to choose the right meridian. But the search should not be limited to the painful meridian and its interior/exterior partner as practised by acupuncturists still at the beginning of their careers. The search for an effective treatment should be more extended to find all treatment possibilities. Further research is necessary to distinguish which system might be most useful for different clinical conditions.

This systematic description of the combinatorial possibilities offers a powerful tool for daily practice. Knowledge of all systems and combination systems helps preserve the balance of the treatment, especially if more than two needles are used in a treatment.

## 5. Conclusion

Following these considerations only two meridians might be excluded as potentially balancing meridians on the basis of TCM theory. For application in treatment further appraisal of the results is necessary. For acupuncture trials most meridians and acupuncture points must be considered as potentially effective, so it seems almost impossible to define noneffective acupuncture points as controls.

## Figures and Tables

**Table 1:** Intrinsic rules of the historical systems.

Every meridian pairs with only one other
Rotation symmetry of 30°, 60°, or 120°
6 pairs of meridians
Maximum of 2 alternating steps
6 yin/yang *or* 3 yin/yin and 3 yang/yang combinations

**Table 2:** Combinations that follow the intrinsic rules of the historical systems, listed according to steps in the Chinese clock.

1 step		2
1 step	3 steps alternating	4
2 steps		4
2 steps	6 steps alternating	4
3 steps		2
5 steps	7 steps alternating	2
6 steps		1

Total number		19

**Table 3:** Possible steps in the Chinese clock for balancing a meridian.

1, 2, and 3 are possible
4 is not possible
5 is possible but has no tradition in TCM, except in the theory
of the extraordinary vessels
6 is possible
